# *Pseudomonas aeruginosa* cells attached to a surface display a typical proteome early as 20 minutes of incubation

**DOI:** 10.1371/journal.pone.0180341

**Published:** 2017-07-05

**Authors:** Marc Crouzet, Stéphane Claverol, Anne-Marie Lomenech, Caroline Le Sénéchal, Patricia Costaglioli, Christophe Barthe, Bertrand Garbay, Marc Bonneu, Sébastien Vilain

**Affiliations:** 1 Spectrométrie de Masse des Macromolécules Biologiques, Chimie Biologie des Membranes et Nanoobjets, UMR CNRS 5248, Institut National Polytechnique de Bordeaux, Université de Bordeaux, Bordeaux, France; 2 Plateforme Protéome, Centre Génomique Fonctionnelle de Bordeaux, Université de Bordeaux, Bordeaux, France; 3 Laboratoire de Chimie des Polymères Organiques, UMR CNRS 5629, Institut National Polytechnique de Bordeaux, Université de Bordeaux, Bordeaux, France; Centre National de la Recherche Scientifique, Aix-Marseille Université, FRANCE

## Abstract

Biofilms are present in all environments and often result in negative effects due to properties of the biofilm lifestyle and especially antibiotics resistance. Biofilms are associated with chronic infections. Controlling bacterial attachment, the first step of biofilm formation, is crucial for fighting against biofilm and subsequently preventing the persistence of infection. Thus deciphering the underlying molecular mechanisms involved in attachment could allow discovering molecular targets from it would be possible to develop inhibitors against bacterial colonization and potentiate antibiotherapy. To identify the key components and pathways that aid the opportunistic pathogen *Pseudomonas aeruginosa* in attachment we performed for the first time a proteomic analysis as early as after 20 minutes of incubation using glass wool fibers as a surface. We compared the protein contents of the attached and unattached bacteria. Using mass spectrometry, 3043 proteins were identified. Our results showed that, as of 20 minutes of incubation, using stringent quantification criteria 616 proteins presented a modification of their abundance in the attached cells compared to their unattached counterparts. The attached cells presented an overall reduced gene expression and characteristics of slow-growing cells. The over-accumulation of outer membrane proteins, periplasmic folding proteins and O-antigen chain length regulators was also observed, indicating a profound modification of the cell envelope. Consistently the sigma factor AlgU required for cell envelope homeostasis was highly over-accumulated in attached cells. In addition our data suggested a role of alarmone (p)ppGpp and polyphosphate during the early attachment phase. Furthermore, almost 150 proteins of unknown function were differentially accumulated in the attached cells. Our proteomic analysis revealed the existence of distinctive biological features in attached cells as early as 20 minutes of incubation. Analysis of some mutants demonstrated the interest of this proteomic approach in identifying genes involved in the early phase of adhesion to a surface.

## Introduction

Bacterial biofilms are a source of recurrent problems in medical fields. Infections caused by biofilms are significant socioeconomic burden that implicates patient diseases, lost employment and hospitalization. In USA, nosocomial infections, mainly related to the presence of biofilms [1,2], cause US$4.5 billion in care surcharge per year and lead to the death of nearly 100,000 inpatients [3]. The origin of these problems lies in the particular physiology of the bacteria immobilized within biofilms, called sessile bacteria, which escape the immune system of the host and are characterized by a “resistant” phenotype [4]. Because current anti-microbial compounds in most cases cannot eradicate biofilms infections, it is therefore a need to develop alternative means to fight against established biofilms and / or prevent their formation. New anti-biofilm strategies require a thorough knowledge about the biology of biofilms. In this context, many studies have been performed on various bacterial species to understand how a biofilm is formed and to identify the genes involved in its development. Consensually, the biofilm development cycle includes reversible and irreversible attachment, microcolony formation, maturation and dispersion [5]. To date, it is difficult to have a precise molecular view of each phase of biofilm formation as the studies are usually conducted on developing or mature biofilms using various growth systems. Bacteria growing in biofilms are physiologically heterogeneous, owing in part to their adaptation to local environmental conditions, depending on their spatial location within the community [6,7]. Regarding the attachment phase, comprehensive studies have been limited to the “late” attachment phase (*i*.*e*. after several hours of incubation) owing to the difficulty of obtaining a sufficient biomass. However, targeted studies (*i*.*e*. mutant analysis) have been performed on the early attachment phase and provide piecemeal information into the molecular players involved in the early stages of biofilm formation [8,9].

Understanding the underlying molecular mechanisms involved in the attachment phase could allow discovering new molecular targets and developing inhibitors against bacterial colonization. With this objective, we have developed an original growth system making it possible to obtain the immobilized biomass needed to perform a comprehensive proteomic analysis as of the first minutes of incubation [10]. In this paper, we present the first comprehensive proteomic analysis of *Pseudomonas aeruginosa* immobilized on glass wool (GW) after only 20 minutes of incubation. *P*. *aeruginosa* is an opportunistic pathogen of medical interest that is responsible for at least 10% of nosocomial infections [11] and which particularly infects immunocompromised hosts suffering from respiratory diseases, cancer and burns as well as cystic fibrosis with an elevated rate of morbidity and mortality [12]. Our analysis consisted of comparing 2 bacterial populations (*i*.*e*. unattached vs attached bacteria) derived from the same growth system and distinguished only by their ability to attach or not to the surface. Comparison of *P*. *aeruginosa* proteomes showed that the physiology of attached bacteria was changed after only twenty minutes of incubation. Among the differentially accumulated proteins between attached cells (AC) and unattached cells (UC), we identified several proteins involved in the early *P*. *aeruginosa* attachment phase on GW.

## Results

### Protein synthesis was required for an optimal colonization of the GW surface

**Fig 1:**
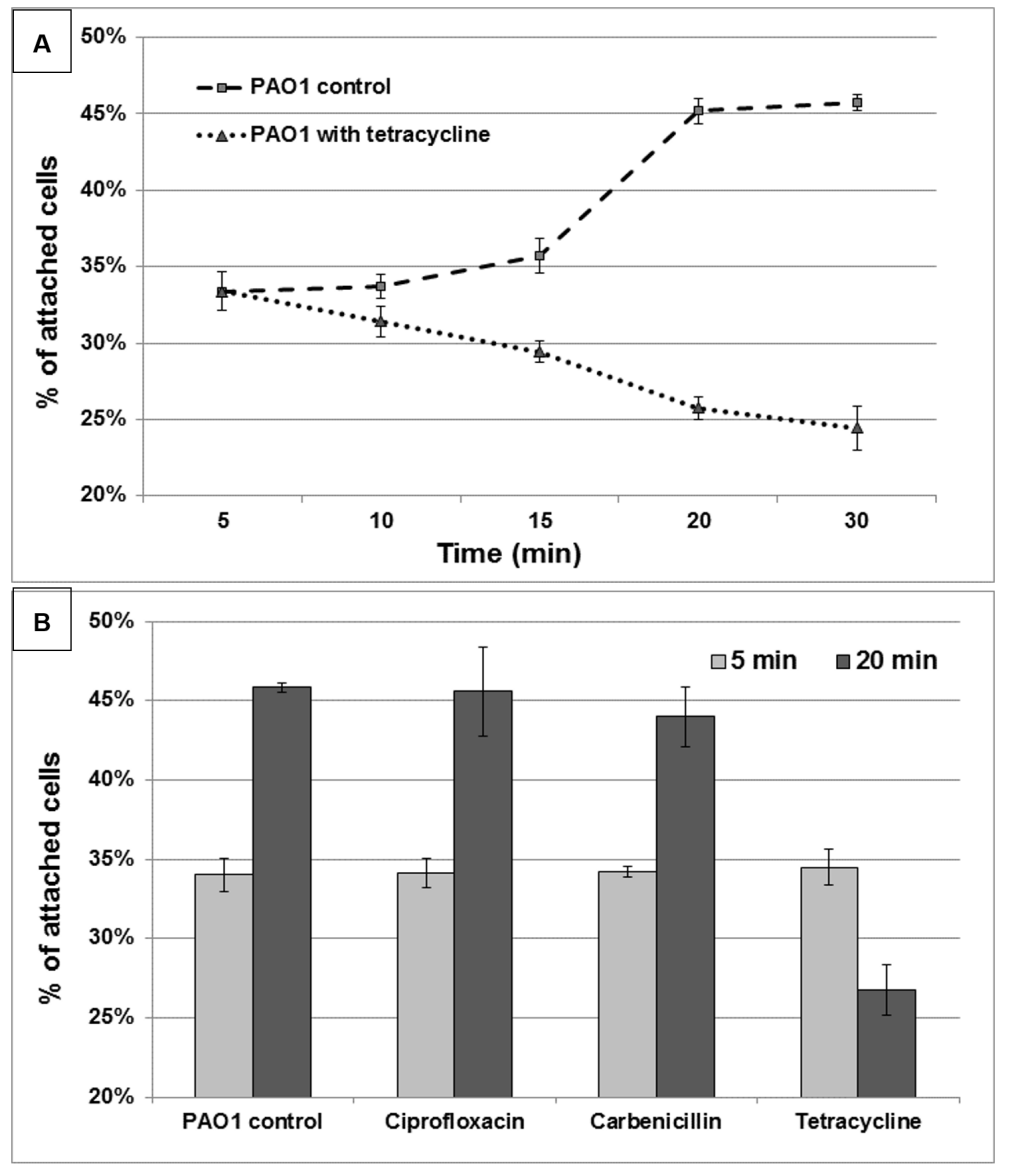
Effect of antibiotics on PAO1 attachment on GW. LB cultures were inoculated at 10^8^ CFU/mL and cells were treated with antibiotics at bacteriostatic concentration. After 5, 10, 15, 20 or 30 min, attached bacteria were recovered and quantified by CFU counting as described in materials and methods. At t0, just after adsorption of the medium onto GW, no attached cells were detected [10]. (**A**) Effect of tetracycline as a function of incubation time up to 30 minutes. (**B**) Comparative analysis of the effect of ciprofloxacin, carbenicillin and tetracycline after 5 and 20 minutes of incubation. Each point is the mean ± SD of biological triplicates.

We previously reported that tetracycline negatively impacts the attachment capacity of PAO1 in both the GW Sponge System and the 96-well plate system [10]. This effect, obtained after 20 min of incubation, led us to conclude that protein synthesis was required for the first step of biofilm formation. Using the same experimental conditions, we performed a time-course analysis of the tetracycline effect and evaluated its specificity. First, the percentage of PAO1 cells that adhered onto GW (AC%) was determined after 5, 10, 15, 20 and 30 min of incubation in the absence or presence of tetracycline (150 μg/mL) (Fig 1A). Up to 15 min, the AC% were similar for cultures with or without antibiotic, even if a slight difference was observed at 15 min. After 20 min, the effect of tetracycline on *P*. *aeruginosa* attachment was marked. Without tetracycline, the AC% increased from 33.4 ± 1.3% to 45.2 ± 0.8% between points 5 and 20 min. With tetracycline, the AC% decreased from 33.4 ± 1.2% to 25.7 ± 0.7%. A similar result was observed after 30 min of incubation (Fig 1A). Secondly, we determined if the observed effect was specific to tetracycline. The experiments described above were performed with carbenicillin and ciprofloxacin at bacteriostatic concentration (200 and 0.4 μg/mL, respectively), 2 antibiotics which unlike tetracycline do not affect protein synthesis [13,14]. Neither carbenicillin nor ciprofloxacin had any effect on the attachment capacity of PAO1 (Fig 1B). This result showed that the reduced PAO1 attachment observed after 20 min in the presence of tetracycline was linked to its ability to inhibit protein synthesis. It also suggested that neosynthesized proteins were necessary for attachment at 20 minutes of incubation. Therefore we decided to perform a comprehensive proteomic analysis after 20 min of incubation.

### Attached and unattached cells already displayed an overall proteome drastically distinct after only 20 minutes incubation

Label-free experiment was performed with 3 biological replicates. 3043 proteins were identified among the 5570 potential proteins of *P*. *aeruginosa*. Among the quantified proteins, 865 proteins exhibited a statistical difference between attached and unattached samples according to Progenesis (ANOVA p-value < 0.05). In the text, these proteins are named "quantified proteins" (S1 Table). From these quantified proteins, the variation between the biological replicates was estimated by calculating the Relative Standard Deviation (RSD) for each protein (S2 Table). Owing to the analyzed variable, RSD included both biological and technical variations within the 3 independent experiments. The mean RSD (± SEM) for AC and UC was 23.2 ± 0.6% and 21.7±0.7%, respectively. The median RSD was 17.7% for AC and 16.0% for UC (S1 and S2 Tables). In addition, the global variation between the biological samples was analyzed via an ANOVA test. Data were Log2 transformed to respect the Normal distribution. The results showed that the distributions were similar (mean, median, maximum, minimun) and, that there was no significant difference between the AC biological replicates (p-value = 0.63) and between the UC biological replicates (p-value = 0.91) (S1 Table). These statistical analyses clearly showed the repeatability of the quantification data between the biological replicates and indicated a low experimental variation between the label-free experiments.

**Fig 2:**
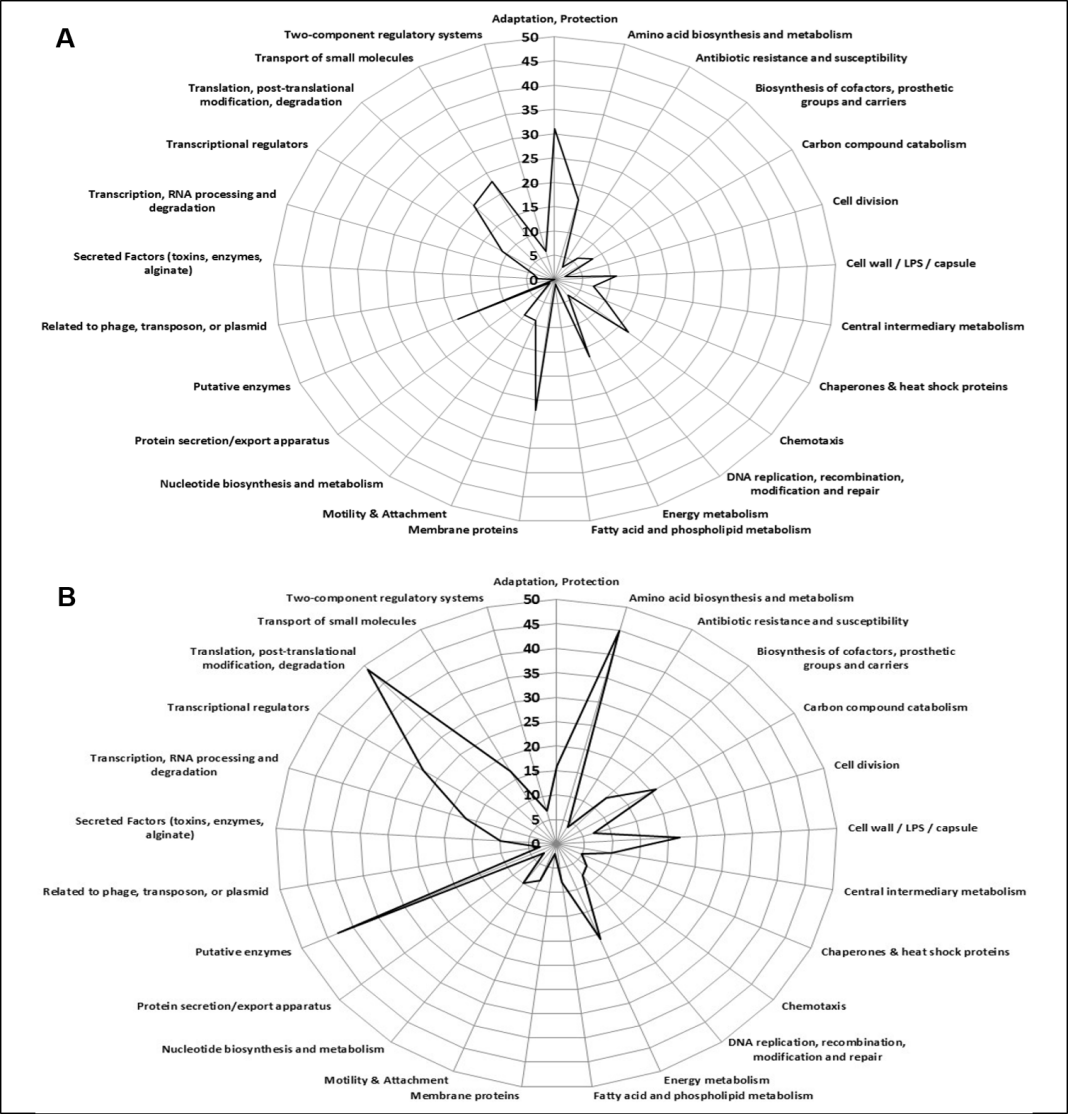
Distribution of differentially accumulated proteins based on functional categories proposed by PseudoCAP database. The graphs show the distribution of over-accumulated (**A**) and under-accumulated (**B**) proteins in attached cells. The hypothetical proteins were not considered in this analysis.

Among the 865 quantified proteins, 616 presented an AC/UC ratio (R) ≥ 2 or ≤ 0.5 (258 over-accumulated and 358 under-accumulated proteins, respectively) and 249 proteins were classified as non-modified (see materials and methods and S2 Table for details). According to the functional categories defined by the PseudoCAP database [15], the over-accumulated proteins in AC mainly belonged to the “adaptation, protection” (31 hits), “membrane proteins” (27 hits) and “chemotaxis” (17 hits) categories (Fig 2A), whereas under-accumulated proteins mainly belonged to the categories related to gene expression: “Transcription, RNA processing and degradation” (17 hits); “transcriptional regulators” (28 hits) and “translation, post-translational modification, degradation” (49 hits) (Fig 2B). Similarly the proteins listed in the “carbon compound catabolism” and in the “biosynthesis of cofactors, prosthetic groups and carriers” categories were rather under-accumulated in AC. This analysis indicated that the AC seemed generally less active than their unattached counterparts. In addition it appeared that the AC metabolism was in part dedicated to adapting to new environmental conditions. These observations were reminiscent of those reporting that low metabolic activity and reduced growth rate are physiological traits of sessile cells in mature biofilms [6]. This overall analysis suggested the existence of specific biological features in AC as early as 20 minutes of incubation. Among the proteins exhibiting a variation in their relative abundance between AC and UC, some likely conferred functional traits allowing *P*. *aeruginosa* cells to initiate colonization on GW. In order to highlight the biological functions involved in the attachment phase, the proteins were grouped according to their defined or predicted functions, the biological pathway or process wherein they are involved, or according to their biochemical characteristics. This clustering was based on data available in the literature and databases (PseudoCAP, COG, KEGG…) and took into account information specific to biofilm formation, such as adhesion, motility, virulence, transport, cell envelope… The protein groups are listed in S3 to S6 Tables and the main results of our investigation are presented below.

### Gene expression and cell growth

We first examined the proteins linked to gene expression as well as cell division in *P*. *aeruginosa*.

#### Gene expression was globally reduced in attached cells

Based on the available information, 56 of the 865 quantified proteins belonged to, or were related to the “transcription, transcriptional regulators and RNA processing and degradation” category (S3A Table). Among them, 7 were over-accumulated and 41 were under-accumulated in AC. Transcription is mediated by a DNA-dependent RNA polymerase and regulated by general factors and transcriptional regulators. The under-accumulation of β and β’ subunits of RNA polymerase (PA4270, R = 0.18 and PA4269, R = 0.11 respectively) suggested that the overall transcriptional process was reduced in AC. Nevertheless NusA (PA4745, R = 2.13) and, more significantly Rnk (PA5274, R = 7.38) and DksA (PA4723, R = 15.77) were over-accumulated in AC. These factors interact with RNA polymerase and are required for the natural progression of the bacterial polymerase [16–17]. Gene expression is also tightly controlled by a repertoire of transcriptional regulators, particularly the sigma factors. 24 sigma factors of which 19 extracytoplasmic function (ECF) were predicted in *P*. *aeruginosa* [18]. In our study, the sigma factors RpoD (PA0576, R = 0.44, σ^70^), RpoS (PA3622, R = 0.12, σ^38^), RpoN (PA4462, R = 0.46, σ^54^) were under-accumulated whereas the ECF AlgU (PA0762, R = 42.91) was highly over-accumulated. Besides sigma factors, PAO1 encodes 434 transcriptional regulators, of which many remain to be characterized [19]. In our study, 15 transcriptional regulators and 11 probable transcriptional regulators were quantified and were mainly under-accumulated suggesting a limited transcriptional activity in AC (S3A Table). The accumulation of MexL (PA3678, R = 3.71), the transcriptional repressor of the mexJK multidrug efflux operon was not in contradiction with this proposal. All these data suggested that the general transcriptional activity was lower in AC compared to their unattached counterparts. The attenuated RNA synthesis activity was corroborated by examining RNA maturation and RNA degradation. 13 proteins involved in RNA maturation, especially in rRNA and tRNA maturation were found, they were all under-accumulated in AC. Among the proteins belonging to the “RNA degradation” category, PA4951 (Orn, R = 3.74) and PA5339 (R = 5.92) were over-accumulated (S3A Table). The protein Orn has a proclivity for degrading RNA of a few nucleotides including 5’-phosphoguanylyl-(3’,5’)-guanosine (pGpG), the hydrolysis product of cyclic diguanylate [20].

To further estimate the gene expression level in AC after 20 min of incubation, the proteins involved in the translation process were analyzed (S3A Table). Remarkably, among the 36 ribosomal proteins quantified, 29 were highly under-accumulated (R ranging from 0.05 to 0.47), indicating that the ribosome abundance was decreased in AC. Our data also indicated that some ribosomal proteins (S1, S6, S8, S10 and L20) were over-accumulated. These proteins could exhibit moonlighting functions and be involved in other metabolic or regulatory aspects apart from ribosome biogenesis [21]. 12 aminoacyl tRNA synthetases and 3 proteins (GatA, GatB and SelA) acting on the aminoacylation process were also quantified. The ratio for these proteins varied from 0.15 to 5.43. The over-accumulation of some tRNA synthetases could in part be linked to the requirements of aminoacyl-tRNAs as biosynthetic precursors and amino acid donors for other macromolecules, outside of translation [22]. With regard to the translation factors, 6 proteins were quantified. The translation initiation factor IF2 (PA4744) which favors the binding of fMet-tRNA^fMet^ to the small ribosomal subunit was under-accumulated (R = 0.37) in AC. It was unclear why the abundance of the initiation factor IF1 (PA2619, working along with IF2) was so high in the AC (R = 11.87). PA4266 and PA2071 encode for FusA1 and FusA2 respectively, 2 elongation factors EF-G [23]. FusA1 was under-accumulated (R = 0.22) while FusA2 was slightly over-accumulated (R = 2.33). Finally, we observed that the ribosome recycling factor (PA3653), which is required for the release of 70S ribosomes from mRNA on reaching the stop codon, was strongly over-accumulated (R = 7.35) in AC. Dissociation of the ribosome following mRNA translation must be promoted in AC. Given the lower abundance of most of the ribosomal constituents and some translation factors, protein synthesis is likely diminished in AC. Altogether, these data suggested an overall reduced transcriptional and translational activity in AC.

#### Growth rate seemed to be limited in attached cells

The proteins related to the “DNA replication, recombination and repair” category are presented in S3B Table. Among the 22 quantified proteins in this category, 12 were under-accumulated and 4 were over-accumulated. The γ/τ (PA1532, R = 0.40) and δ (PA2961, R = 0.28) subunits of DNA polymerase III, the main enzyme involved in the replication of the bacterial genome, were under-accumulated in AC. These subunits are part of the minimal clamp loader for loading the β subunit necessary for the high processivity of DNA polymerase III [24]. In addition PA4931 encoding for the replicative DnaB helicase was greatly under-accumulated (R = 0.04). Accordingly we postulated that DNA replication is less efficient in AC. 5 nucleoid-associated proteins playing a central role in global chromatin organization [25] were quantified. The MksE (PA4685, R = 2.68) and ScpB (PA3197, R = 3.35) condensins were over-accumulated while ScpA (PA1527, R = 0.20) and Hu (PA1804, R = 0.22) were under-accumulated in AC. Interestingly, the Dps protein (PA0962), a DNA-binding protein from starved cells, was over-accumulated (R = 2.83) [26]. These results suggested a particular organization of bacterial chromatin in AC. 8 proteins of the “DNA recombination and repair” category were quantified (S3B Table), of which 5 were under-accumulated and none over-accumulated in AC. The abundance level of these proteins was consistent with the hypothesis of a reduced chromosomal DNA replication process as a whole.

Quantitative data related to the proteins involved in the “Cell division” and the “Cell wall peptidoglycan synthesis” categories are also presented in S3B Table. Cell division is orchestrated by FtsZ and more than 10 essential cell division Fts proteins [27]. In our study, FtsZ (PA4407, R = 2.37) was slightly over-accumulated in AC while FtsA (PA4408) was non-modified and FtsE (PA0374, R = 0.16), involved in peptidoglycan modification, was under-accumulated. We also observed an important over-accumulation of MinE (PA3245, R = 8.39), a membrane-bound ATPase, resulting in the concentration of MinC/MinD complex being the lowest at mid-cell. MinC anchored to the plasma membrane through binding to MinD prevents FtsZ polymerization at the poles [28]. The under-accumulation of MinD (PA3244, R = 0.22) suggested a delay in cell division. Furthermore, the relative abundance of most of the quantified proteins involved in peptidoglycan synthesis or remodeling was reduced in AC; only PonA and DacC were over-accumulated (S3B Table). All these data suggested that, at 20 min of incubation, AC growth was limited compared to that of UC.

### Signaling

Bacteria use signaling pathways to translate external signals into adaptive responses at the functional level. We examined two-component and chemosensory systems as well as second messenger systems described to be implicated in the switch between planktonic and biofilm modes of growth [9].

#### Two-component and chemosensory systems

Biofilm formation is controlled by a wide range of factors, of which two-component systems (TCS) play a major role [29]. Among the 10 two-component response regulators quantified, 6 were under-accumulated in AC (S4A Table), including AlgR (PA5261, R = 0.12) and PilR (PA4547, R = 0.31) which are involved in alginate production and type IV pilus biogenesis respectively. CbrB (PA4726, R = 0.43), which belongs to the CbrAB/Crc system needed for optimizing energy utilization, was also under-accumulated in AC. In addition to TCS, *P*. *aeruginosa* contains 4 chemosensory pathways that mediate chemotaxis, flagella motility, type IV pili-based motility, and alternative cellular functions [30,31]. The chemotaxis gene clusters PA1456-PA1464 and PA3348-PA3349 are required for flagella motility in *P*. *aeruginosa* [32]. For this signaling pathway, only the CheY response regulator (PA1456, R = 13.30) and the chemotaxis protein CheZ (PA1457, R = 2.22) were over-accumulated in AC while the other quantified proteins were non-modified or under-accumulated (S4A Table). The relative abundance of CheA (PA1458, R = 0.42) which mediates signaling from a chemoreceptor to CheY was reduced in AC, suggesting that this pathway was less active in AC. In agreement with a reduced signal transduction CheR1, a methyltransferase (PA3348, R = 0.23) and CheB, a methylesterase (PA1459, R = 0.40) which control methylation of the chemoreceptor were under-accumulated in AC. Moreover the over-accumulation of CheZ which inactivates CheY by dephosphorylation may be part of this hypothesis. The Chp chemosensory pathway PA0408-PA0417 indirectly impacts type IV pili biosynthesis/twitching motility as a result of a primary defect in cAMP production [33]. Twitching motility allows *P*. *aeruginosa* to respond to stimuli by extending and retracting its type IV pili. The sensory protein component PilJ (PA0411, R = 8.87) as well as the CheY homologs PilG (PA0408, R = 4.45) and PilH (PA0409, R = 9.17) were over-accumulated in AC. Concerning PilG and PilH, it was *a priori* surprising because they differentially regulate the activity of the CyaB adenylate cyclase; PilG activates cAMP synthesis whereas PilH inhibits it. However the PilJ over-accumulation suggested that the Chp chemosensory system was more active in AC. The *che* cluster II genes PA0173-PA0180 are required for an optimal chemotactic response [34]. This pathway seemed also more active in AC in so far as the methyl-accepting chemotaxis protein CttP/McpA (PA0180, R = 17.82) and the sensor CheA2 (PA0178, R = 2.25) were over-accumulated. Consistently the relative abundance of CheB2 (PA0173) methylesterase was lower in AC (R = 0.11). Finally the *wsp* genes contained in the cluster PA3702-PA3708 have been proposed to contribute to bacterial biofilm formation [35]. The Wsp pathway responds to solid surfaces and WspA, a membrane-bound receptor protein, detects a signal associated with growth on a surface [36]. The increase in WspA abundance (PA3708, R = 2.51) in AC was consistent with an activation of this pathway in these cells. Furthermore, additional methyl-accepting chemotaxis proteins (MCPs) mediate signal transduction to chemosensory systems; MCPs sense the stimuli that initiate the chemotactic activity [37]. 26 MCPs were inventoried from *P*. *aeruginosa* genome. In addition to WspA and CttP/McpA, 10 MCPs were quantified (S4A Table). Many of them were over-accumulated in AC indicating a chemotaxis contribution on the initial attachment process.

#### Second messenger systems

Second messenger systems are critical for regulating transition from planktonic to biofilm mode of growth [38–40]. c-di-GMP is involved in the transition between the planktonic and the sessile states [9]. In our analysis, the proteins related to c-di-GMP LapD (PA1433, R = 8.49) and PA2567 (R = 2.18) were over-accumulated in AC (S4B Table). LapD favored cell aggregation in the presence of an increased c-di-GMP level and inactivation of PA1433 reduced attachment [41,42]. Furthermore as the attached *P*. *aeruginosa* presented characteristics of slow-growing cells, we explored the proteins involved in production of the (p)ppGpp alarmone and subsequently in stringent response. In *P*. *aeruginosa* the level of (p)ppGpp is controlled by enzymes belonging to the RelA and SpoT family [43]. RelA which synthesizes (p)ppGpp was over-accumulated in AC (PA0934, R = 2.15; S4B Table); this data suggested an increase of (p)ppGpp amount during cell attachment as of 20 min of incubation. Interestingly the relative abundance of the polyphosphate kinase Ppk1 (PA5242, R = 7.18) was strongly elevated in AC. Ppk1, a major enzyme in polyphosphate production, is controlled by (p)ppGpp level [44]. For comparison the polyphosphate kinases (PA0141, R = 0.27 and PA3455, R = 0.43) belonging to the Ppk2 family were under-accumulated in AC.

### Outer membrane components and appendages

Several surface-associated adhesion molecules including pili, flagella, outer membrane proteins, exopolysaccharides and lipopolysaccharides mediate the attachment of *P*. *aeruginosa* to biotic and abiotic surfaces. The quantified proteins related to these components are listed in S5 Table.

#### Pili and flagella

Type IV pili are involved in adherence, motility and pathogenesis [45,46]. Based on our data, it was difficult to establish a clear picture of the contribution of type IV pili in *P*. *aeruginosa* during the attachment phase (S5A Table). The relative abundance of structural components as PilA, PilO and PilQ was not modified while that of PilN and PilU was under-accumulated in AC. In addition, the inner membrane protein FimV required for type IV pilus assembly and twitching motility [47] was over-accumulated (PA3115, R = 3.73). The same heterogeneity was observed for the proteins related to flagella (S5A Table). However the master regulator FleQ which controls transcription of structural and regulatory genes involved in flagella synthesis was under-accumulated in AC (PA1097, R = 0.31). This observation suggested that flagella production in AC could be reduced after 20 min of incubation with GW. Additionally we also noted the under-accumulation of Pfm (PA2950, R = 0.45). Rotation of the flagellar filament and consequently cell swimming is powered through the proton motive force [48]; the lower Pfm abundance in AC could be related to the recently acquired immobilized state of the cells. At last MotB (PA4953, R = 3.25), a component of the MotAB stator, was over-accumulated. The MotAB stator may play a role in modulating irreversible attachment on abiotic surfaces [49].

#### Outer membrane proteins

Most OMPs are surface-exposed so they are potentially important in interfacing bacteria with abiotic and biotic surfaces [50]. In our study, 12 OMPs were quantified (S5B Table). Interestingly most of them were over-accumulated in AC. Among them, OprI (PA2853, R = 9.07) was shown to adhere to the epithelial cells of the trachea and the small intestine of chickens [51]. OprH (PA1178, R = 2.71) is thought to increase the stability to the outer membranes of *P*. *aeruginosa* by directly interacting with lipopolysaccharide. Edrington *et al*. [52] provided evidence for multiple interactions between OprH and lipopolysaccharide that likely contribute to the antibiotic resistance of *P*. *aeruginosa*. The over-accumulation of OMPs indicates that AC might respond to the attachment onto GW surface by outer membrane changes as of 20 min of incubation.

#### Exopolysaccharides

Mature biofilm cells are embedded in an extracellular polymeric substance matrix composed of polysaccharides, extracellular DNA, lipids and/or proteins. *P*. *aeruginosa* is known to produce at least 3 different polysaccharides involved in biofilm development—alginate, Psl and Pel. In the PAO1 strain, Psl contributes to attachment on glass while Pel plays no role in this phase [53]. For Psl synthesis, PslF (PA2236, R = 0.39) and PslI (PA2239, R = 0.28) were under-accumulated while the relative abundance of PslE (PA2235) was increased by a factor 12 in AC (S5C Table). PslE is predicted to act as the polysaccharide copolymerase [54], and could affect polysaccharide chain length in AC. None of the 13 proteins involved in the alginate biosynthesis pathway was listed in the quantified proteins. The AlgZR TCS controls alginate biosynthesis and is essential for alginate production [55]. In our analysis AlgR (PA5261, R = 0.12, S4A Table) was under-accumulated, indicating a restricted alginate synthesis in AC.

#### Lipopolysaccharides

The proteins involved in lipopolysaccharide (LPS) biosynthesis are presented in S5C Table. LPS are essential components of the *P*. *aeruginosa* cell envelope. These molecules comprise a lipid A portion, an oligosaccharide core and a highly variable polysaccharide known as the O-antigen. LPS physical properties such as the 3D-structure and the number of repeating units contribute to bacterial adhesion [56,57]. When looking at lipid A data, LpxL2 (PA0011) was under-accumulated (R = 0.02) while LpxO2 (PA0936) was highly over-accumulated (R = 41.80) in AC. LpxL2 was proposed to transfer the secondary lauroyl groups to lipid A and LpxO2 might hydroxylate one of the two secondary acyl chains [58]. These data suggested that *P*. *aeruginosa* lipid A could adopt different chemical modifications in response to early attachment onto GW. Concerning the oligosaccharide core, the common polysaccharide antigen and B-band O-antigen, the proteins were rather under-accumulated in AC. However, for the O-specific antigen, the length regulators Wzz1 (PA3160; R = 14.97) and Wzz2 (PA0938; R = 4.83) were over-accumulated in AC. The Wzz proteins control the length of the O-antigen polymer by determining the number of subunits added [59]. In PAO1, modal chain lengths of 12–16 and 22–30 repeat units are conferred by Wzz1 while the Wzz2 protein is responsible for the very long O-antigen chain length (40–50 repeat length). Longer LPS molecules might be one answer for strengthening the attachment to the glass surface. Moreover WpbM (PA3141) was strongly over-accumulated in AC (R = 48.52). This UDP-4,6-GlcNAc dehydratase is essential for O-antigen synthesis in many serotypes of *P*. *aeruginosa* [60]. These results were consistent with the hypothesis that the AC rapidly modified their envelope as suggested above from the OMP analysis.

### Biofilm physiology

The features of bacteria within mature biofilms (>24h) frequently described in the literature are adaptation to microenvironments [61], setting up of an intercellular communication [62], increased virulence [63] and an outstanding resistance to biocides [64]. We examined some of these traits in AC at 20 min of incubation (S6 Table).

#### Environmental stress response and protein folding

Bacterial adhesion leads to changes in the cell envelope and stress responses within biofilms are activated [65,66]. To explore stress response following GW attachment, molecular chaperones as well as cold- and heat-shock proteins mainly involved in protein folding are listed (S6A Table). Most of the chaperones were over-accumulated (ClpB, DnaK, GroEL, GroES, GrpE, HptG and HscA) in AC, only DnaJ and the trigger factor were under-accumulated. DnaK and GroEL are major ubiquitous chaperones that play crucial roles in promoting protein folding [67]. DnaJ is one of 3 co-chaperones that interacts with DnaK. The trigger factor interacts with polypeptides early during ongoing synthesis and its under-accumulation (PA1800, R = 0.47) could be linked to a reduced translation level in AC. Remarkably the cold-induced proteins listed in S6A Table were all strongly over-accumulated in AC. In comparison, the relative abundance of the universal stress proteins Usp was not modified. The cold-induced proteins play a role in the adaptation of *P*. *aeruginosa* to stresses through transcriptional as well as translational regulation [68], and could also be involved in the attachment process. The peptidyl-prolyl isomerases (PPIases) were also added to this part of the study (S6A Table). PPIases catalyze prolyl *cis*-*trans* isomerization and facilitate proper protein folding [69]. *P*. *aeruginosa* isolated from cystic fibrosis patients overexpressed PPIases [70]. Among the quantified proteins, 3 PPIases were over-accumulated in AC, particularly PpiD (PA1805, R = 13.79) and PpiC2 (PA4176, R = 26.83). Altogether these data highlighted the importance of protein folding and chaperoning during the attachment of PAO1 to GW.

#### Quorum sensing, virulence and resistance

Quorum sensing (QS), a bacterial cell-to-cell communication, is involved in the expression of virulence factors such as extracellular proteases, efflux pump expression and biofilm formation [71,72]. *P*. *aeruginosa* possesses 3 QS systems (*las*, *rhl* and PQS) which drive the production of homoserine lactones (HSL: 3-oxo-C12-HSL and C4-HSL) and 2-heptyl-hydroxy-quinolone (PQS). No proteins involved in HSL synthesis were listed in the quantified proteins. PqsD (PA0999) and PqsH (PA2587) were listed in the non-modified class (S6B Table) suggesting that quinolone-based QS was similar in AC and UC after 20 min incubation. We also observed the under-accumulation of RhlA (PA3479, R = 0.20), a protein involved in the production of rhamnolipids and in the swarming motility [73], whose gene is regulated by *rhlR*. This under-accumulation may be related to the under-expression of the *rhl* system in the AC. Furthermore we noted that the secreted virulence factors AprA (PA1249), LasA (PA1871), LasB (PA3724), Piv (PA4175) as well as PA0572 (a metalloprotease) were clearly under-accumulated in AC (R ranging from 0.03 to 0.32). These virulence factors were shown to be regulated by the Las QS system. In addition, we observed the under-accumulation of Vfr (PA0652, R = 0.06; S3A Table), a virulence transcriptional regulator known to positively control *lasR* [39]. Apart from proteases controlled by QS, we also observed the under-accumulation of other proteases such as CtpA (PA5134, R = 0.24), Lon (PA1803, R = 0.09), PepA (PA3831, R = 0.26) and PA3787 (R = 0.02) (S6B Table). Altogether our data suggested that neither the *las* system nor the *rhl* system are necessary for early attachment of *P*. *aeruginosa* in the GW Sponge System, as well as for a number of proteases.

Efflux of antibiotics by Resistance-Nodulation-Cell Division (RND) pumps is considered the major factor responsible for high antibiotic resistance [74]. To date, 11 different RND pumps capable of effluxing antibiotics/antimicrobial products have been characterized in *P*. *aeruginosa*. Members of the MexAB-OprM, MexGHI-OpmD and TriABC-OpmH efflux pumps were quantified (S6B Table). MexB (PA0426, R = 18.55) and MexI (PA4207, R = 5.86) were over-accumulated in AC while the other members of these efflux pumps had an R ranging from 1.57 to 1.86 (S6B Table). The proteins constituting the MexEF-OprN pump were not quantified but 2 factors regulating this system were. MexT (PA2492), a transcription factor, activates the genes of the MexEF-OprN system while MexS (PA2491), an oxidoreductase, has been described as an inhibitor [75]. MexT was under-accumulated (R = 0.17) whereas MexS was over-accumulated (R = 3.23), suggesting that *mexEF-oprN* genes were less expressed in AC after 20 min. PA1874 was also over-accumulated (R = 2.80). This protein is part of an efflux pump (PA1874-PA1877) involved in biofilm-specific resistance to certain antibiotics in PA14 [76]. The over-accumulation of some elements of RND pumps suggest that AC could be more resistant to antibiotics than their UC counterparts after only 20 minutes of incubation.

### Analysis of the highest differentially accumulated proteins in attached cells

To complete our study, we focused on the highest differentially accumulated proteins in AC compared to their UC counterparts. For this, only proteins whose relative quantity was increased by at least a factor 10 (*i*.*e*. 57 proteins) or decreased by one log or more (*i*.*e*. 80 proteins) were selected (S7 Table). Observing so many highly differentially accumulated proteins (137) early as 20 minutes of incubation with GW reinforced the hypothesis of the need for a particular physiology dedicated to the attachment. Among these proteins, 33 proteins have an unknown function and 29 have a probable function according to the Pseudomonas Genome Database [15]. Because of this high proportion of proteins of unknown function, we first focused on the known interactions between the proteins listed in S7 Table by using the software STRING v.10 [77] with the default setting. Among the 80 strongly under-accumulated proteins, 55 were predicted to interact (S1A Fig). In this category, ribosomal proteins were grouped together, as expected, as well as several proteins related to virulence. In addition, the analysis predicted an interaction between PA5133 and PA3787, 2 conserved hypothetical proteins both related to metallopeptidase according to the COG database. Among the 57 strongly over-accumulated proteins, 31 were predicted to be interactants (S1B Fig). The analysis predicted 2 groups comprising 2 interacting proteins (PA0270-PA0565; PA3202-PA5229) all of unknown function. The software STRING also revealed interactions on the one hand between 2 proteins belonging to the P-type ATPase (PA1429-PA3920), both probably involved in the transport of cation and, on the other hand between the proteins PA5055 and PA5065 (UbiB). Additionally, by rummaging *P*. *aeruginosa* PAO1 Protein Interactome [78], we found a relevant interaction between PA0950 and PA3664 both linked to oxidation-reduction process. All these proteins with large variations of relative abundance between AC and UC could be part of pathways determinant for surface attachment. Moreover, as protein synthesis appeared diminished in AC, the study of strongly over-accumulated proteins could be of particular interest for identifying proteins involved in the attachment phase.

**Fig 3:**
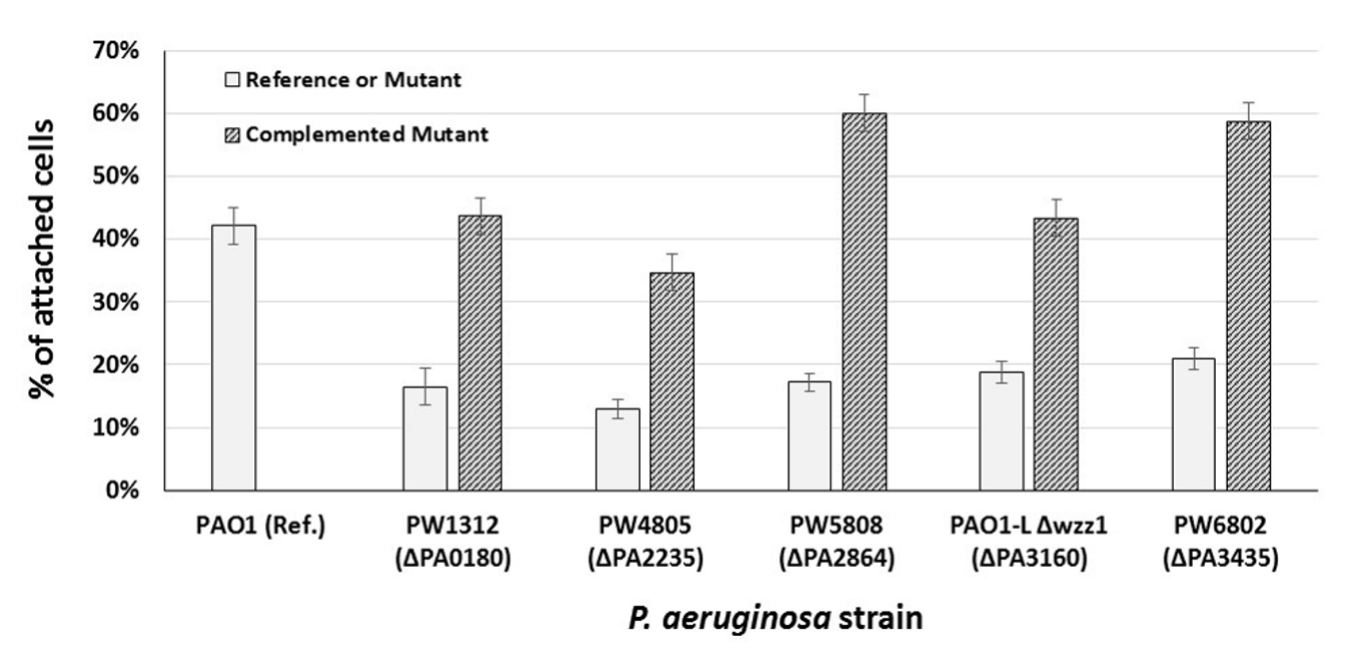
Attachment capacity of *P*. *aeruginosa* PAO1 reference strains and isogenic mutants. The attachment capacity of reference strains, mutants and complemented mutants (see S9A Table) was assayed after 20 min at 37°C in our glass wool system (see materials and methods). Only the attachment capacity of *P*. *aeruginosa* PAO1 was presented for reason of clarity, the other reference strains (PAO1-L and MPAO1) showed results similar to those obtained for PAO1. In the same manner, the attachment capacities of the different mutants were not altered by introducing the empty plasmid pUCP20 or pUCP22. For each of the mutants presented herein, the complemented strain displayed an attachment capacity ranging from 80% to 140% of the reference strain (see S8 Table). The results corresponded to the average of 3 independent experiments (bar = SD).

Validation of our proteomic data was carried out by testing the attachment capacity of available mutants inactivated for genes corresponding to the highly over-accumulated proteins in AC. Among the 43 mutants analyzed, 8 strains showed a marked difference in attachment ability compared to PAO1 control (S8 Table). 3 of them (PA0591, PA0950 and PA3675) presented an increase in the attachment capacity (ratio 1.5 to 1.6). For the others (PA0180, PA2235, PA2864, PA3160 and PA3435) there is a large decrease in attachment ability (ratio ranging from 0.3 to 0.5), indicating that these genes play an important role in the adhesion process as early as 20 minutes of incubation. When complemented, the latter 5 mutants attached at levels comparable to the reference strain after 20 minutes of incubation (Fig 3). This provided genetic evidence that these genes are required for attachment.

## Discussion

For the first time, by using our experimental approach, we have thrown light on the protein content of *P*. *aeruginosa* during the very early stage of attachment. Even if we did not quantify all *P*. *aeruginosa* proteins, our analysis provided an overview of what can biologically happen and highlighted some features about mechanisms underlying bacterial attachment. The comparison of the protein content of the attached and unattached cells cultured under the same experimental conditions and the use of stringent quantification criteria, strengthened the reliability of our data. In addition, performing analysis after a very short time of incubation should reduce the cell heterogeneity of the attached bacterial population, which is problematic in the interpretation of the results of comprehensive analysis performed on mature biofilms. Our analysis demonstrated that after 20 minutes of incubation there were already strong changes in protein abundance in number and magnitude in the AC. These results showed that the AC expressed a specific protein profile indicative of either phenotypic adaptations following immobilization and/or the existence of a molecular pre-equipment for colonization. After 20 minutes of incubation, the AC presented a reduced gene expression and protein markers characteristics of slow-growing cells. This observation is reminiscent of the general view that sessile cells within mature biofilms are slow-growing and metabolically little active [79]. Nevertheless, we noted that the general transcription factor DksA was over-accumulated in AC. DksA, which is involved in a variety of cellular processes, including cell division, stringent response and quorum sensing [80], could play a major role in transcription control in the particular physiology of AC. DksA potentiates the effects of (p)ppGpp on transcription [43]. We also noticed that after attachment RelA, the enzyme that synthesises the alarmone (p)ppGpp, was over-accumulated suggesting an elevated concentration of this nucleotide in AC. The over-accumulation of the main polyphosphate synthetic enzyme Ppk1, which has a central role in gene regulation and responds to elevated (p)ppGpp [44], was also consistent with this hypothesis. Additionally, (p)ppGpp is known to be involved in biofilm formation [81,82] and a *ppk1* mutant of the PAO1 strain is defective in biofilm formation [83]. Our data suggest that through proteins like DksA and Ppk1, the alarmone (p)ppGpp may play a key role in the process of attachment to a surface as of 20 min of incubation. This interpretation is fully consistent with the results of Hancock’s team dissecting the inhibitory role of peptides on the formation of bacterial biofilm [84,85]. They identified peptides which potently prevent biofilm formation by triggering the degradation of (p)ppGpp in bacteria. Following all these observations, we may suppose that the attachment capacity of a bacterium requires a physiological state at least based on a reduced overall gene expression associated with an increased concentration of (p)ppGpp and polyphosphate. As part of this physiological response, Ppk1 would also be a good target for the design of inhibitors anti-biofilm. Indeed far no Ppk1 homologue has been identified in higher-order eucaryotes and deletion of the *ppk1* gene in bacterial pathogens results in a defective survival and loss of virulence towards protozoa and animals [86].

Another characteristic of AC was a strong increase in AlgU abundance. AlgU may be a key factor during cell attachment. This extracytoplasmic function sigma factor (σ^22^) is known to modulate the cell wall response to stress and outer envelope biogenesis and remodeling [87]. It was reported that the AlgU stimulon overlaps with biofilm control mechanisms. When the 293 genes in the AlgU stimulon were compared to those activated during biofilm development more than half of the genes were in common [87]. The over-accumulation of chemotaxis receptors, outer membrane proteins, periplasmic chaperones, PPIases and other membrane proteins is in agreement with cell envelope modifications. Altogether our data suggested that fine-tuning of envelope quality controls constitutes an important layer of regulation in AC. Regarding virulence, the c-AMP dependent transcriptional regulator Vfr was under-accumulated in AC. Vfr promotes the expression of multiple acute virulence factors and is determinant for pathogenesis and for biofilm formation during bacterial infection [88]. In our *in vitro* system the virulence factors controlled by Vfr were not necessary for attachment to GW. This demonstrates that the attachment phase can be separated from the virulence physiology associated to the Vfr regulator. Apart from the proteins mentioned above, many other proteins also displayed differential abundance between UC and AC. Some are involved in redox system, electron transfer and energy source while others have been reported to be involved in biofilm formation such as LecA (PA2570) [89], PstS (PA5369) [90] and PA3731 [91].

Another feature of AC was the existence of a pool of highly over-accumulated proteins. Our mutant analysis allowed identifying genes involved in the attachment phase. Among these genes we identified PA2235 (*pslE*) already shown to be necessary for *P*. *aeruginosa* attachment [92], and PA0180 (*cttP/mcpA*) involved in the maturation of biofilms [93]. This analysis also demonstrated for the first time the involvement in the early attachment stage of PA3160 (*wzz1*), PA3435 encoding a flavodoxin and PA2864 encoding a protein of unknown function. Interestingly, for 3 mutant strains (PA0591, PA0950 and PA3675) a net increase in the attachment capacity was observed (ratio 1.5 to 1.6). No clear function is attributed to these genes but we can hypothesize that these genes negatively control mechanisms involved in early attachment. Our results showed the interest of this proteomic approach in identifying genes involved in the early phase of adhesion to a surface.

During this work, we also found that GW colonization was reduced in the presence of tetracycline, although some bacteria were still capable of attaching to the fibers. This could be due to the fact that tetracycline was not immediately effective and required time to penetrate and achieve an inhibitory concentration for protein synthesis. During this time, one part of the bacterial population could adhere to the surface. However, it was reported that the rate of tetracycline uptake was rapid. In *Pseudomonas putida* tetracycline was accumulated in excess of the external concentration within 1 min of incubation and tetracycline accumulation was 10-fold in excess of the external concentration after 15 min of incubation [94]. Likewise, tetracycline uptake in non-proliferating *E*. *coli* suspension was observed within 3 min incubation with antibiotic [95]. These data suggested that tetracycline was effective quickly after its addition to the *P*. *aeruginosa* culture. Furthermore, we have noticed that preincubation of *P*. *aeruginosa* cells with tetracycline for 30 min before the addition of GW provided a similar proportion of AC without preincubation. Another explanation may be that a sub-population already present in the inoculum possessed the molecular equipment required for attachment. Therefore, this sub-population would be unaffected by an antibiotic targeting protein synthesis because the proteins needed for attachment would already be present in the bacteria. To verify the existence of this sub-population, another protein analysis would need to be performed for a shorter incubation time, which our experimental system allows. The existence of a molecular pre-equipment should result in the presence of a common pool of proteins differentially accumulated at 20 min and within a shorter time. The comparison of the 2 analyses could then identify the key components of the attachment phase.

From our data, it is obvious that the mechanism of initial attachment to a surface is a complex process involving numerous factors. The proteome dataset reported here will serve as a resource for detailed investigations of surface attachment in *P*. *aeruginosa*. Among these proteins, novel anti-biofilm molecular targets could be discovered. *In vitro* biofilm studies have provided valuable explanations for early events in biofilm infections [96]. In addition, as AC already exhibited characteristics of slow-growing bacteria after only 20 min, our analysis reinforces the need to develop new inhibitory molecules other than antibiotics that are generally effective on dividing cells.

## Materials and methods

### Strains, culture conditions and molecular constructions

All the strains used in this study are presented in S9A Table. *Pseudomonas aeruginosa* PAO1 (CIP 104116) was provided by the Institut Pasteur (France); *P*. *aeruginosa* MPAO1 was provided by the Seattle *P*. *aeruginosa* PAO1 transposon mutant library and Pr. Lam provided another strain of *P*. *aeruginosa* PAO1 named PAO1-L. All strains were grown in Lennox Lysogeny Broth (LB) supplemented when necessary with the appropriate antibiotic. All cultures were performed at 37°C under agitation (150 rpm). Overnight pre-cultures were obtained by inoculating 20 mL of LB with one isolated bacterial colony on LB agar plate. The Glass Wool (GW) Sponge System used to study cell attachment was previously described [10]. Our system is based on the adsorption of a culture medium onto GW fibers, using the high retention capacity of GW, like a sponge adsorbs a liquid. Briefly, calibrated pieces of GW (Sodipro Company, Echirolles, France, ref. number SCI03950) were sterilized in water and dried after sterilization. The inoculum was prepared by diluting the pre-culture as appropriate in fresh LB, and then 5 mL were adsorbed onto 1g of GW.

The mutant strains were obtained from the *P*. *aeruginosa* PAO1 transposon mutant library whose the quality was checked [97]. The strain inactivated for PA3160 (Δ*wzz1*) was gently provided by Pr. Lam [59]. To carry out the molecular constructions described below polymerase and enzymes were all provided and used as recommended by New England BioLabs (NEB) company. To complement the PA0180, PA2864, PA3160 and PA3435 transposon mutants, the corresponding genes with their own promoter and terminator were amplified by PCR from the PAO1 genomic DNA using the Q5^®^ High-Fidelity DNA Polymerase and primers listed in S9B Table. The amplified fragments digested by EcoRI and HindIII were cloned into EcoRI/HindIII-digested pUCP20 [98] and transformed into NEB^®^ 5-alpha Competent *Escherichia coli*. Transformants were selected onto LB agar containing 100 μg/mL ampicillin. For the PA2235 gene, included in an operon, only the coding sequence bordered by the EcoRI and HindIII sites was amplified by PCR and inserted into pUCP22 under the control of the *lacZ* promoter also functional in *P*. *aeruginosa*. Transformants were selected onto LB agar containing 15 μg/mL gentamycin. The resultant plasmids were verified by DNA sequencing. The recombinant plasmids or the control empty plasmids pUCP20 or pUCP22 were transferred by chemical transformation into the corresponding *P*. *aeruginosa* mutants and transformants were selected onto LB agar containing 200 μg/mL of carbenicillin or 125 μg/mL gentamycin.

### Antibiotics effect on *P*. *aeruginosa* PAO1 attachment

The kinetic study of the tetracycline effect on PAO1 attachment was performed as described previously [10]. Briefly, the inoculum was prepared by diluting the pre-culture in fresh LB at 1/100, corresponding to ≈10^8^ CFU/mL and the tetracycline concentration was adjusted to 150 μg/mL. Then 5 mL of the bacterial suspension were adsorbed on GW and incubated at 37°C. After 5, 10, 15, 20 or 30 min, GW was washed with PBS (NaCl 8 g/L, KCl 0.2 g/L, Na_2_HPO_4_ 2H_2_O 1.44 g/L, KH_2_PO_4_ 0.24 g/L) and the attached and unattached bacteria were quantified by CFU counting [10]. The number of CFU was determined by plating twice 100 μL of serial dilutions of bacterial suspensions onto LB agar and incubating for 24h at 37°C. All time points were performed in biological triplicate. Similarly, the effects of carbenicillin, ciprofloxacin and tetracycline on the attachment of PAO1 onto GW were compared after 5 and 20 min of incubation. The carbenicillin and ciprofloxacin (Sigma-Aldrich) concentrations used were 200 and 0.4 μg/mL, respectively, corresponding to 2 fold the minimal inhibitory concentration. To determine the bacteriostatic effect at these concentrations, a bacterial suspension was treated for 1h with each antibiotic and plated on LB agar for CFU counting. 3 independent experiments were performed.

### Bacterial attachment assays

The attachment capacity of *P*. *aeruginosa* PAO1 reference strains, mutants (containing or not pUCP20 or pUCP22) and complemented mutants was determined after 20 minutes of incubation by using our GW system [10]. All overnight pre-cultures were obtained as described above. The inoculum was prepared by diluting the pre-culture to 1/100 in fresh LB, and then 5 mL were adsorbed onto 1g of GW for bacterial attachment assays. After 20 minutes of incubation at 37°C (agitation 150 rpm), attached and unattached cells were recovered and quantified by colony forming unit (CFU) counting as previously described [10]. 3 independent experiments were performed.

### Preparation of attached and unattached biomasses by using the glass wool sponge system

The biomasses of attached cells (AC) and unattached cells (UC) were obtained using our system [10]. Briefly, bacterial inoculum was prepared by diluting a pre-culture in fresh LB at 1/10, corresponding to ≈10^9^ CFU/mL and 5 mL were adsorbed onto a 1g GW piece. After 20 min of incubation, UC were harvested by collecting 3 mL of the liquid adsorbed on GW with a 5 mL pipette. This sample was immediately used for protein extraction. Then this GW piece was placed in a 50 mL syringe and washed with 100 mL PBS running down through the GW by gravity so eliminating the residual UC from GW. PBS on GW was straightaway removed by pipetting leaving ≈ 3 mL on the surface. Then GW was directly treated to extract proteins from the AC. The protocol and schemes illustrating bacteria surrounding GW are presented in S2 Fig. The UC and AC samples were prepared from 3 biological replicates.

### Protein extraction

Our system allows starting protein extraction from UC and AC (directly on fibers) within 40 seconds without excessive handling of the samples [10]. A rapid cell lysis should avoid gene expression changes that result from the cell harvesting process. Bacteria were lysed by adding 1 volume of lysis solution 2X (7 M urea, 2 M thiourea, 65 mM CHAPS, 20 mM DTT, 1 M NaCl) to 1 volume of sample (*i*.*e*. UC or the piece of GW carrying the AC). The mix was frozen (-80°C, 30 min) and thawed (34°C, 20 min). The extracted proteins were concentrated by a classical 15% (v/v) TCA precipitation followed by 2 acetone washings. Proteins were suspended in 50 μL of the following solution: 7 M urea, 70 mM SDS, 20 mM DTT. 9 μL of protein extract were mixed with 3 μL of Laemmli buffer 4X and then 10 μL were loaded on SDS-PAGE (10%). After electrophoresis, proteins were visualized by colloidal Coomassie blue staining. The gels were scanned with a GS-800 densitometer (BioRad) and analyzed by QuantityOne software (BioRad) to determine the sample volumes so as to have the same quantity of proteins for mass spectrometry studies.

### Label-free analysis: Experimental design for protein identification and quantification

#### Sample preparation

Before the label-free analysis, protein samples were decomplexified on SDS-PAGE (10%). The 6 biological samples to be compared (UC 1–3; AC 1–3) were loaded on the gel with a protein quantity estimated at 30 μg per sample. Protein separation was stopped when the migration front reached the bottom of the resolving gel. After colloidal blue staining, each lane was divided into 4 parts of equal length and each part was cut into 1 mm x 1 mm gel pieces. The gel pieces were destained in 25 mM ammonium bicarbonate 50% ACN, rinsed twice in ultrapure water and shrunk in ACN for 10 min. After ACN removal by pipetting, gel pieces were dried at room temperature, covered with the trypsin solution (10 ng/μL in 40 mM NH_4_HCO_3_ and 10% ACN), rehydrated at 4°C for 10 min, and finally incubated overnight at 37°C. The gel pieces were then incubated for 15 min in 40 mM NH_4_HCO_3_ and 10% ACN at room temperature with rotary shaking. The supernatant was collected, and an H_2_O/ACN/HCOOH (47.5:47.5:5) extraction solution was added onto the gel pieces for 15 min. The extraction step was repeated twice. The pooled supernatants were dried in a vacuum centrifuge and then resuspended in 40 μL of water acidified with 0.1% HCOOH. Peptide samples were stored at -20°C.

#### nLC-MS/MS analysis

Peptide mixture was analyzed on an Ultimate 3000 nanoLC system (Dionex, Amsterdam, The Netherlands) coupled to an Electrospray Q-Exactive quadrupole Orbitrap benchtop mass spectrometer (Thermo Fisher Scientific, San Jose, CA). Ten microliters of peptide digests were loaded onto a 300-μm-inner diameter x 5-mm C18 PepMapTM trap column (LC Packings) at a flow rate of 30 μL/min. The peptides were eluted from the trap column onto an analytical 75-mm id x 15-cm C18 Pep-Map column (LC Packings) with a 4–40% linear gradient of solvent B in 108 min. Mobile phases were a mix of solvent A (0.1% formic acid in 5% ACN) and solvent B (0.1% formic acid in 80% ACN). The separation flow rate was set at 300 nL/min. The mass spectrometer operated in positive ion mode at a 1.9-kV needle voltage. Data were acquired using Xcalibur 2.2 software in a data-dependent mode. MS scans (m/z 300–2000) were recorded at a resolution of R = 70000 (@ m/z 200) and an automatic gain control (AGC) target of 1 x 10^6^ ions collected within 100 ms. Dynamic exclusion was set to 30 s and the top 15 ions were selected from fragmentation in higher-energy collisional dissociation (HCD) mode. MS/MS scans with a target value of 1 x 10^5^ ions were collected with a maximum fill time of 120 ms and a resolution of R = 35000. Additionally, only +2 and +3 charged ions were selected for fragmentation. Other settings were as follows: neither sheath nor auxiliary gas flow; heated capillary temperature, 200°C; normalized HCD collision energy of 25% and an isolation width of 3 m/z.

#### Database search and results processing

Data were searched by SEQUEST through Proteome Discoverer 1.4 (Thermo Fisher Scientific Inc.) against the protein database from the Pseudomonas Genome Database website [15] consisting of 5570 entries. Spectra from peptides higher than 5000 Da or lower than 350 Da were rejected. The search parameters were as follows: mass accuracy of the monoisotopic peptide precursor and peptide fragments was set to 10 ppm and 0.02 Da, respectively. Only b- and y-ions were considered for mass calculation. Oxidation of methionine (+16 Da), deamidation of asparagine and glutamine (+1 Da), propionamide (+71 Da) and carbamidomethylation of cysteine (+57 Da) were considered as variable modifications. Two missed trypsin cleavages were allowed. Peptide validation was performed using the Percolator algorithm [99] and only “high confidence” peptides were retained corresponding to a 1% false positive rate at peptide level.

#### Label-free quantitative data analysis

Raw LC-MS/MS data were imported in Progenesis LC-MS 4.1 (Nonlinear Dynamics Ltd, Newcastle, UK). Data processing included the following steps: (i) Ion detection: the most sensitive feature detection setting was chosen and to reduce noise, features displaying chromatographic peaks with width lower than 0.04 minute were removed, (ii) Automatic features alignment was carried out across all samples, (iii) Peak picking was performed on all data files leading to the absence of missing values. Volumes were integrated for 2–6 charge-state ions, (iv) Raw data were normalized to a reference data file based on ratio median calculated from LC-MS features, (v) Peptide and protein IDs from Proteome Discoverer filtered to a false discovery rate set at 1% were imported, (vi) An ANOVA test was performed at peptide level to eliminate unsignificant differential abundance features (p > 0.05), (vii) Protein abundance in each sample was calculated by summing volumes of all unique peptides, (viii) An additional ANOVA test was conducted at protein level and the proteins with p > 0.05 were not retained for biological analysis. Quantitative data were considered for proteins quantified by a minimum of 2 peptides and only proteins fulfilling all the criteria mentioned above from the set of identified proteins were included in the final quantification lists. The mass spectrometry proteomics data have been deposited to the ProteomeXchange Consortium [100] via the PRIDE partner repository with the dataset identifier PXD002942.

### Comparison of the relative protein abundance between attached and unattached cells

Progenesis selected proteins that were statistically differentially accumulated between AC and UC [see S1 Table]. The quantitative values of each protein in AC and UC were used to calculate the AC/UC ratio. This ratio indicated the direction and magnitude of the variation of the relative protein abundance between AC and UC. Based on a factor 2, already used in proteomic studies [101], 3 different classes were defined. A protein was considered as “over-accumulated” (OVER) in AC if AC/UC ratio was ≥ 2.0, “under-accumulated” (UNDER) in AC if AC/UC ratio was ≤ 0.5. Proteins with an AC/UC ratio ranging from 0.5 to 2.0 were classified as “non-modified” (NM). For the sake of clarity, the ratio AC/UC is referred to as R in the text.

## Supporting information

S1 Fig Protein interactions for highly differentially accumulated proteins. Interactions were determined using the software STRING v.10 with the default setting. Proteins whose relative quantity was decreased in attached cells by one log or more (*i*.*e*. 80 proteins, **A**) or increased by at least a factor 10 (*i*.*e*. 57 proteins, **B**) were analyzed. Lines indicate protein-protein interactions. Color code and default setting are available on STRING website (http://string-db.org/).
(DOCX)

S2 Fig Harvesting of attached and unattached cells from the glass wool (GW) Sponge System. A resume of the protocol used to harvest attached and unattached cells is illustrated in the left part of the figure. The location of bacteria after each step is shown schematically in the corresponding right part. First (**Box 1**), 5 mL of bacterial inoculum (≈10^9^ CFU/mL) were adsorbed onto a 1g GW piece. The culture medium formed a homogeneous layer surrounding the fibers. After 20 min of incubation (**Box 2**), unattached cells (**blue cells**) were harvested with a 5 mL pipette. The cells were immediately lysed and the lysate used for protein extraction. Finally (**Box 3**), the residual unattached and slightly attached cells (**yellow cells**) were eliminated. The GW was placed in a 50 mL syringe and washed by 100 mL PBS running through the GW by gravity. The remaining PBS on GW was removed straightaway by pipetting. Attached cells (**green cells**) on the GW were directly treated for protein extraction.
(DOCX)

S1 Table Summary of the label-free experiment results and reliability of quantitative data. ^(■)^ Among the quantified proteins, 865 proteins exhibited a statistical difference between attached and unattached samples according to Progenesis (ANOVA p-value < 0.05) (**Part 1**). In the text, these proteins are named "quantified proteins". The reliability of these data was estimated by the Relative Standard Deviation (RSD) (**Part 2**) and an ANOVA test (**Part 3**). RSD evaluates the variation of the quantitative data of a protein between the biological replicates. The ANOVA test estimates the variation between the quantified proteomes of each biological replicate. In addition, the distribution of the quantified proteomes was visualized using a box-and-whisker plot (**Part 3**). The 865 quantified proteins were distributed in the OVER, UNDER and NM classes according to the AC/UC ratio (**Part 4**).
(DOCX)

S2 Table List of identified and quantified proteins. (XLSX)

S3 Table Quantitative information of proteins related to gene expression and cell growth. (XLSX)

S4 Table Quantitative information of proteins related to signaling. (XLSX)

S5 Table Quantitative information of proteins related to outer membrane components and appendages. (XLSX)

S6 Table Quantitative information of proteins related to biofilm physiology. (XLSX)

S7 Table List of the highest differentially accumulated proteins in attached cells. (XLSX)

S8 Table Attachment capacity of *P*. *aeruginosa* mutants inactivated for genes corresponding to the highly over-accumulated proteins. The attachment capacity of the different strains (see S9A Table for strains description) was assayed as described in materials and methods. The results corresponded to the mean (± SD) of 3 independent experiments. (**1**) Only the attachment capacity of *P*. *aeruginosa* PAO1 was indicated as the other reference strains showed results similar to those obtained for PAO1. ^(◆)^ According http://www.pseudomonas.com/.
(DOCX)

S9 Table List of the strains and primers used in this study. (XLSX)
