# Supplementary figures and images for: Pseudomonas aeruginosa cells attached to a surface display a typical proteome early as 20 minutes of incubation
Source: PLoS One. 2017 Jul 5;12(7):e0180341. doi: 10.1371/journal.pone.0180341 (PMC5498041; doi:10.1371/journal.pone.0180341)

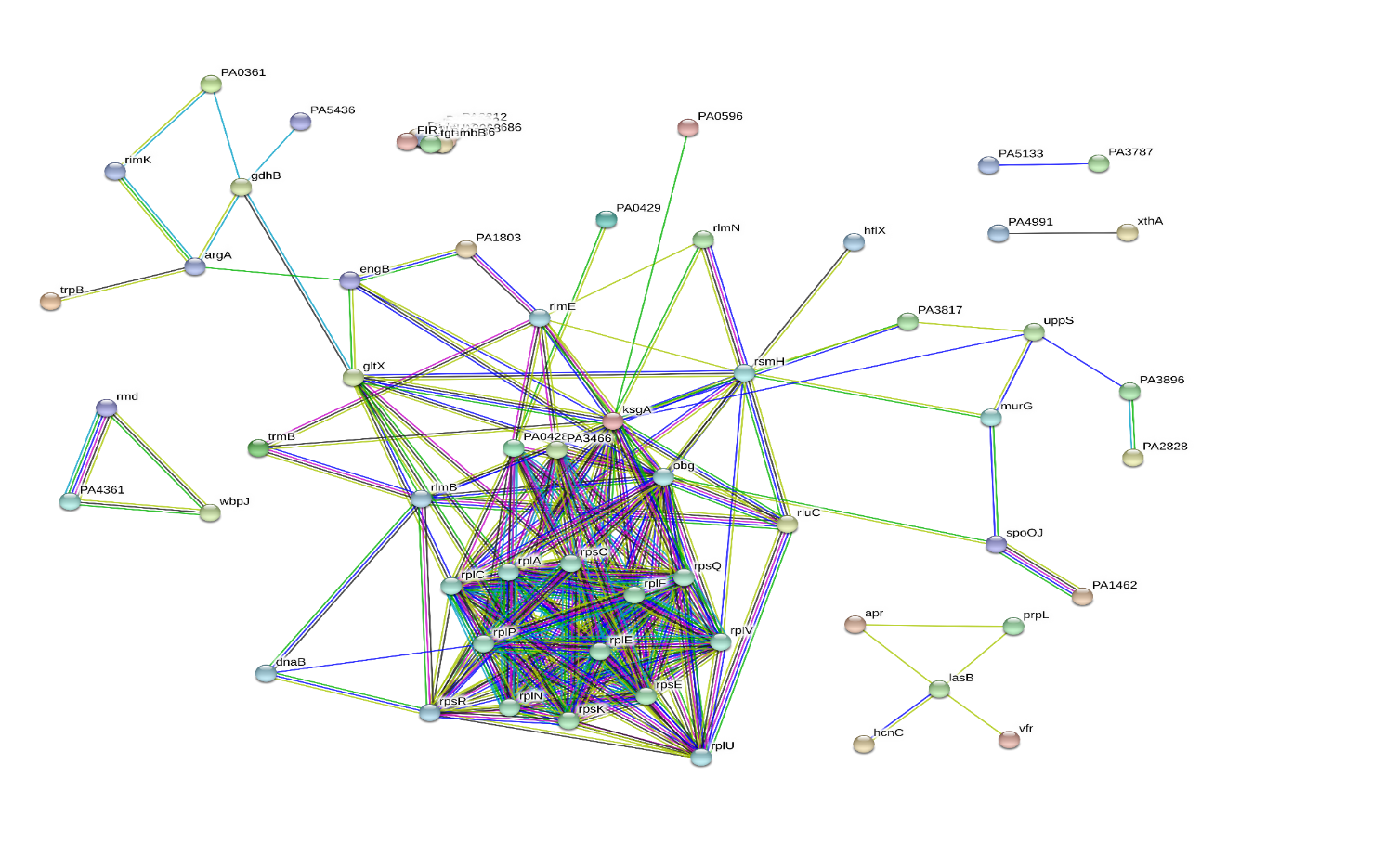


***Ribosomal proteins***

***Virulence***

**A**

**B**


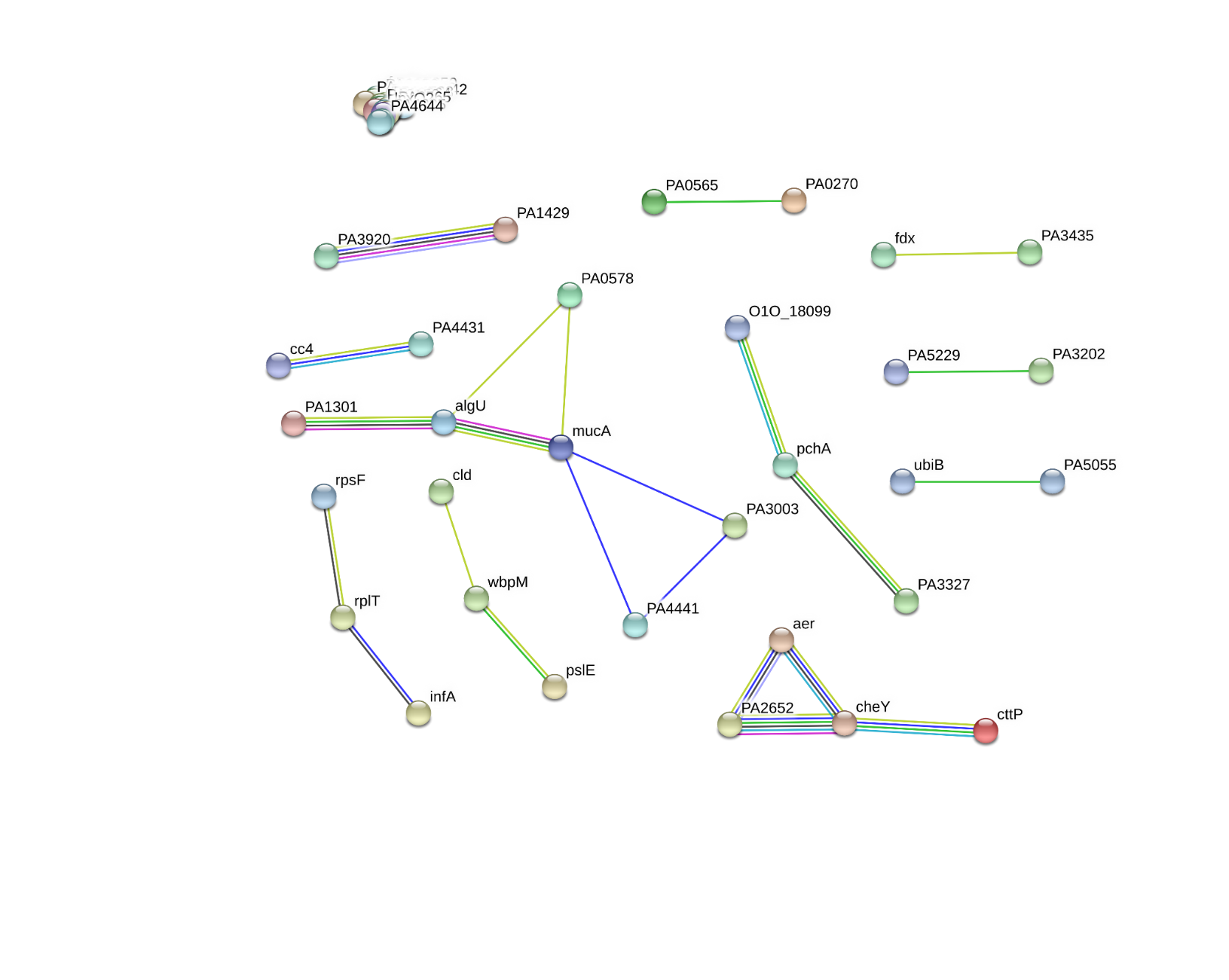

Supplement: S1 Fig — Interactions were determined using the software STRING v.10 with the default setting. Proteins whose relative quantity was decreased in attached cells by one log or more (i.e. 80 proteins, A) or increased by at least a factor 10 (i.e. 57 proteins, B) were analyzed. Lines indicate protein-protein interactions. Color code and default setting are available on STRING website (http://string-db.org/). (DOCX) [file pone.0180341.s001.docx]

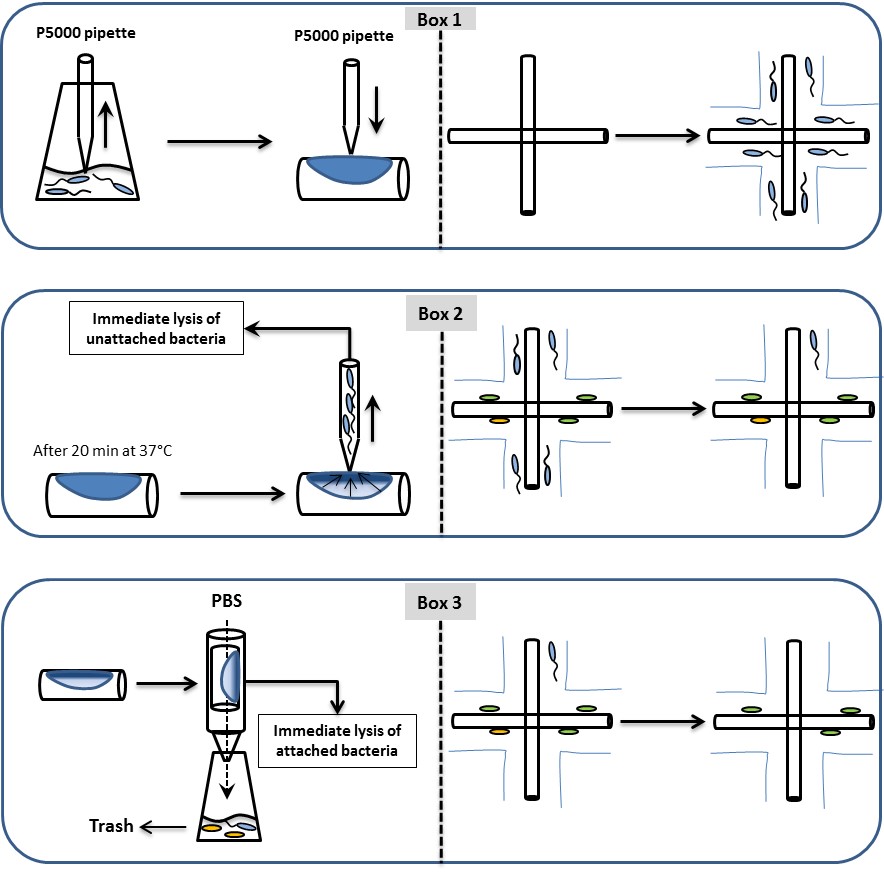

Supplement: S2 Fig — A resume of the protocol used to harvest attached and unattached cells is illustrated in the left part of the figure. The location of bacteria after each step is shown schematically in the corresponding right part. First (Box 1), 5 mL of bacterial inoculum (≈109 CFU/mL) were adsorbed onto a 1g GW piece. The culture medium formed a homogeneous layer surrounding the fibers. After 20 min of incubation (Box 2), unattached cells (blue cells) were harvested with a 5 mL pipette. The cells were immediately lysed and the lysate used for protein extraction. Finally (Box 3), the residual unattached and slightly attached cells (yellow cells) were eliminated. The GW was placed in a 50 mL syringe and washed by 100 mL PBS running through the GW by gravity. The remaining PBS on GW was removed straightaway by pipetting. Attached cells (green cells) on the GW were directly treated for protein extraction. (DOCX) [file pone.0180341.s002.docx]
