# Supplementary material for: Pseudomonas aeruginosa cells attached to a surface display a typical proteome early as 20 minutes of incubation
Source: PLoS One. 2017 Jul 5;12(7):e0180341. doi: 10.1371/journal.pone.0180341 (PMC5498041; doi:10.1371/journal.pone.0180341)
Supplement: S1 Table — (■) Among the quantified proteins, 865 proteins exhibited a statistical difference between attached and unattached samples according to Progenesis (ANOVA p-value < 0.05) (Part 1). In the text, these proteins are named "quantified proteins". The reliability of these data was estimated by the Relative Standard Deviation (RSD) (Part 2) and an ANOVA test (Part 3). RSD evaluates the variation of the quantitative data of a protein between the biological replicates. The ANOVA test estimates the variation between the quantified proteomes of each biological replicate. In addition, the distribution of the quantified proteomes was visualized using a box-and-whisker plot (Part 3). The 865 quantified proteins were distributed in the OVER, UNDER and NM classes according to the AC/UC ratio (Part 4). (DOCX) [file pone.0180341.s003.docx]

| **Part 1. Identified and quantified proteins from the label-free experiment.** | | | |
| --- | --- | --- | --- |
| Peptides identified |  |  | 29505 |
| Proteins identified |  |  | 3043 |
| Proteins quantified and statistically differentially accumulated ^(■)^ | |  | 865 |

| **Part 2. Reliability of data - Relative Standard Deviation (expressed as a %).** UC = Unattached Cells; AC = Attached Cells; SEM = Standard error of the mean. | | |
| --- | --- | --- |
| Samples | (Mean ± SEM) | Median |
| *UC 1, 2 & 3* | *21.7 % ± 0.7 %* | *16.0 %* |
| *AC 1, 2 & 3* | *23.2 % ± 0.6 %* | *17.7 %* |

| **Part 3. Reliability of data – ANOVA test and box-and-whisker plot.** As data followed a Log-Normal distribution (Q-Q plot), data were Log2 transformed to respect the Normal distribution of the data, then an ANOVA test was performed (α = 0.05). The AC or UC distributions were similar (mean, median, maximum, minimum) and there was no significant difference between the AC or UC biological replicates. |
| --- |


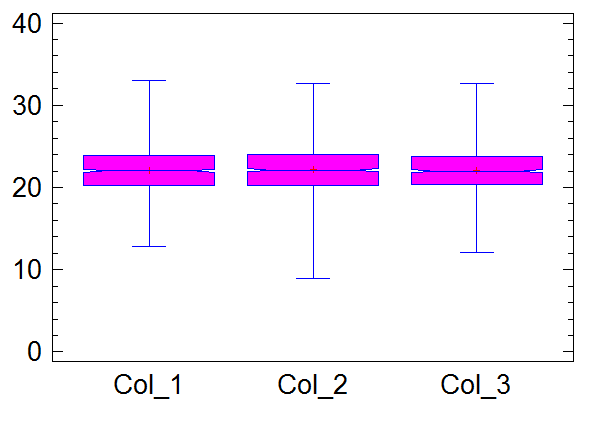

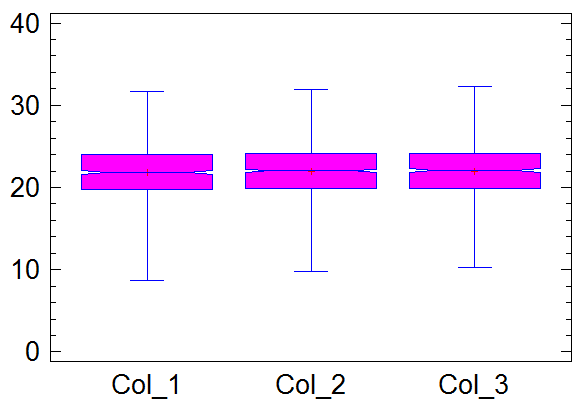


**UC1**

**UC2**

**UC3**

**AC1**

**AC2**

**AC3**

**Log2 Normalized abundance**

**Log2 Normalized abundance**

MIN

MAX

MEAN

MEDIAN

MIN

MAX

MEAN

MEDIAN

ANOVA

ANOVA

12.9

33.1

22.1

22.0

9.0

32.6

22.1

22.1

12.1

32.7

22.1

22.0

p-value = 0.91

8.7

31.7

21.8

21.9

9.8

31.9

22.0

22.0

10.3

32.3

22.0

22.1

p-value = 0.63

| **Part 4. Distribution of quantified proteins in classes.** A protein was considered as “over-accumulated” (OVER) in AC if AC/UC ratio was ≥ 2.0, “under-accumulated” (UNDER) in AC if AC/UC ratio was ≤ 0.5. Proteins with an AC/UC ratio ranging from 0.5 to 2.0 were classified as “non-modified” (NM). | | | | | |
| --- | --- | --- | --- | --- | --- |
|  | **OVER** |  | **258** |  |  |
|  | **UNDER** |  | **358** |  |  |
|  | **NM** |  | **249** |  | **Total: 865** |
