# Supplementary material for: Pseudomonas aeruginosa cells attached to a surface display a typical proteome early as 20 minutes of incubation
Source: PLoS One. 2017 Jul 5;12(7):e0180341. doi: 10.1371/journal.pone.0180341 (PMC5498041; doi:10.1371/journal.pone.0180341)
Supplement: S8 Table — The attachment capacity of the different strains (see S9A Table for strains description) was assayed as described in materials and methods. The results corresponded to the mean (± SD) of 3 independent experiments. (1) Only the attachment capacity of P. aeruginosa PAO1 was indicated as the other reference strains showed results similar to those obtained for PAO1. (◆) According http://www.pseudomonas.com/. (DOCX) [file pone.0180341.s010.docx]

| **Strain** | **Interrupted ORF ^(⧫)^** | **Attachment capacity** | **Mutant / PAO1 ratio** |
| --- | --- | --- | --- |
| *P. aeruginosa* PAO1 **^(1)^** | Reference strain | 42.9% ± 2.9% | 1.0 |
| PW4805 | PA2235 | 13.0% ± 1.5% | 0.3 |
| *PW4805 /C* | *Complemented mutant* | *34.7% ± 2.9%* | *0.8* |
| PW1312 | PA0180 | 16.5% ± 2.9% | 0.4 |
| *PW1312 /C* | *Complemented mutant* | *43.6% ± 0.6%* | *1.0* |
| PW5808 | PA2864 | 17.2% ± 1.4% | 0.4 |
| *PW5808 /C* | *Complemented mutant* | *60.0% ± 3.6%* | *1.4* |
| PAO1-L ∆*wzz*1 | PA3160 | 18.8% ± 1.8% | 0.4 |
| *PAO1-L ∆wzz1 /C* | *Complemented mutant* | *43.4% ± 1.5%* | *1.0* |
| PW6802 | PA3435 | 21.0% ± 1.7% | 0.5 |
| *PW6802 /C* | *Complemented mutant* | *58.8% ± 1.8%* | *1.4* |
| PW2702 | PA0936 | 30.3% ± 2.1% | 0.7 |
| PW3438 | PA1341 | 30.3% ± 3.1% | 0.7 |
| PW6362 | PA3202 | 31.5% ± 3.2% | 0.7 |
| PW8422 | PA4395 | 35.1% ± 2.4% | 0.8 |
| PW7625 | PA3920 | 36.1% ± 3.5% | 0.9 |
| PW8079 | PA4176 | 36.6% ± 2.5% | 0.9 |
| PW1489 | PA0270 | 37.7% ± 2.7% | 0.9 |
| PW2387 | PA0763 | 38.1% ± 3.2% | 0.9 |
| PW5440 | PA2652 | 39.7% ± 2.0% | 0.9 |
| PW6029 | PA3003 | 39.8% ± 2.8% | 0.9 |
| PW3596 | PA1429 | 40.1% ± 3.2% | 1.0 |
| PW4173 | PA1805 | 41.1% ± 2.8% | 1.0 |
| PW1894 | PA0486 | 41.5% ± 3.1% | 1.0 |
| PW7480 | PA1887 | 41.8% ± 3.1% | 1.0 |
| PW6600 | PA3327 | 42.3% ± 2.6% | 1.0 |
| PW7196 | PA3664 | 42.4% ± 2.7% | 1.0 |
| PW8181 | PA4231 | 42.4% ± 3.1% | 1.0 |
| PW9720 | PA5184 | 43.0% ± 2.1% | 1.0 |
| PW8463 | PA4431 | 43.4% ± 1.7% | 1.0 |
| PW7408 | PA3785 | 43.5% ± 1.9% | 1.0 |
| PW8511 | PA4464 | 43.7% ± 1.9% | 1.0 |
| PW9208 | PA4880 | 43.7% ± 1.9% | 1.0 |
| PW7150 | PA3621 | 44.9% ± 3.8% | 1.1 |
| PW3811 | PA1561 | 45.3% ± 2.5% | 1.1 |
| PW2040 | PA0565 | 45.7% ± 2.3% | 1.1 |
| PW9144 | PA4842 | 45.7% ± 3.4% | 1.1 |
| PW6646 | PA3352 | 45.9% ± 1.9% | 1.1 |
| PW4221 | PA1833 | 46.2% ± 2.7% | 1.1 |
| PW10287 | PA5490 | 46.5% ± 3.3% | 1.1 |
| PW8828 | PA4644 | 46.8% ± 2.2% | 1.1 |
| PW8409 | PA4387 | 47.2% ± 4.1% | 1.1 |
| PW3354 | PA1301 | 48.3% ± 2.8% | 1.1 |
| PW4727 | PA2184 | 48.3% ± 3.3% | 1.1 |
| PW1781 | PA0426 | 49.5% ± 3.5% | 1.2 |
| PW2064 | PA0578 | 51.8% ± 4.8% | 1.2 |
| PW2084 | PA0591 | 62.3% ± 4.2% | 1.5 |
| PW7215 | PA3675 | 65.0% ± 4.9% | 1.5 |
| PW2728 | PA0950 | 68.4% ± 3.4% | 1.6 |
